# Stigma towards patients with schizophrenia and other mental disorders: Challenges and interventions in Italy

**DOI:** 10.1192/j.eurpsy.2021.91

**Published:** 2021-08-13

**Authors:** B. Carpiniello

**Affiliations:** Italian Psychiatric Association; Secretary Of The Epa-council Of Npas, Department of Medical Sciences and Public Health University of Cagliari, Cagliari, Italy

## Abstract

Stigma toward mental illness is considered a major public health problem, being a significant obstacle for the access to care by people with psychiatric disorders, not only the severe ones but also those improperly called “minor” disorders, as recent research demonstrates. Moreover, stigma per se causes further sufference, undermining the quality of life of those who suffer from mental disorders due to discrimination, social isolation and lack of opportunities. Thus, combating stigma is one of the main goal of mental health policies worldwide. After the 1978 Reform Act, substantial ideological and practical changes were introduced in Italy, such as, among others, the abandonement of custodial care and of the dangerousness criterion for involuntary treatments, along with the development of a nationwide system in mental health care. Notwithstanding there relevant changes and more than forty years of experience in community treatment of mental disorders and the widespread implementation of interventions oriented to social inclusion, no data about significant changes in public stigma toward mentally ill people could be registered in our country. Moreover, a quite limited number of specific anti-stigma programmes and campaigns at a national or local level were developed with a correspondently paucity of research regarding the evaluation of these interventions with specific reference to their quality and effectiveness

**Disclosure:**

No significant relationships.

